# Exploring Gender Differences in Coding at the Beginning of Primary School

**DOI:** 10.3389/fpsyg.2022.887280

**Published:** 2022-09-20

**Authors:** Chiara Montuori, Lucia Ronconi, Tullio Vardanega, Barbara Arfé

**Affiliations:** ^1^Department of Developmental Psychology and Socialization, University of Padova, Padova, Italy; ^2^School of Psychology, University of Padova, Padova, Italy; ^3^Department of Mathematics, University of Padova, Padova, Italy

**Keywords:** gender gap, coding, STEM, Computer Science, computational thinking, executive function, primary school

## Abstract

The gender gap in Computer Science (CS) is widely documented worldwide. Only a few studies, however, have investigated whether and how gender differences manifest early in the learning of computing, at the beginning of primary school. Coding, seen as an element of Computational Thinking, has entered the curriculum of primary school education in several countries. As the early years of primary education happen before gender stereotypes in CS are expected to be fully endorsed, the opportunity to learn coding for boys and girls at that age might in principle help reduce the gender gap later observed in CS education. Prior research findings however suggest that an advantage for boys in coding tasks may begin to emerge already since preschool or the early grades of primary education. In the present study we explored whether the coding abilities of 1st graders, at their first experience with coding, are affected by gender differences, and whether their presence associates with gender differences in executive functions (EF), i.e., response inhibition and planning skills. Earlier research has shown strong association between children's coding abilities and their EF, as well as the existence of gender differences in the maturation of response inhibition and planning skills, but with an advantage for girls. In this work we assessed the coding skills and response inhibition and planning skills of 109 Italian first graders, 45 girls and 64 boys, before an introductory coding course (pretest), when the children had no prior experience of coding. We then repeated the assessment after the introductory coding course (posttest). No statistically significant difference between girls and boys emerged at the pretest, whereas an advantage in coding appeared for boys at the posttest. Mediation analyses carried out to test the hypothesis of a mediation role of EF on gender differences in coding show that the gender differences in coding were *not* mediated by the children's EF (response inhibition or planning). These results suggest that other factors must be accounted for to explain this phenomenon. The different engagement of boys and girls in the coding activities, and/or other motivational and sociocognitive variables, should be explored in future studies.

## Introduction

With gender differences, or gap, in Science, Technology, Engineering, and Mathematics (STEM) researchers refer to the disparity between boys and girls, or men and women, in performance, achievements, interests or beliefs in the STEM domains. As these gender disparities lead to an underrepresentation of women in higher STEM education and careers (Wang and Degol, 2017), their emergence is considered of high theoretical and practical (societal) relevance. Gender differences in STEM have been addressed extensively from secondary school onwards (Fisher and Margolis, 2003; Zweben and Aspray, 2004; Frieze, 2005; Anderson et al., 2008; Maloney et al., 2012; Spearman and Watt, 2013; Beyer, 2014; Charles et al., 2014; Charlesworth and Banaji, 2019; Alonso et al., 2021; Gnambs, 2021). Comparatively less studies, instead, have investigated the emergence of early gender differences in preschool or elementary school (e.g., Cvencek et al., 2011; Aesaert and van Braak, 2015; Kersey et al., 2018; Master et al., 2021). In this paper we address this particular angle of the problem, considering the emergence of gender-ability differences in the learning of coding and programming at school entrance, in grade one.

Researchers have offered two main explanations to the emergence of gender differences in STEM. The first explanation maintains that the gender gap originates from (innate) sex-differences in the cognitive abilities underpinning performance in STEM (Halpern and LaMay, 2000; Miller and Halpern, 2014; Girelli, 2022). Sex-related differences in cognitive abilities underpinning performance in STEM are indeed reported in some studies (Halpern and LaMay, 2000; Maloney et al., 2012; Miller and Halpern, 2014). These gender disparities can determine differences in students' achievements (Maloney et al., 2012) and consequently affect motivation for the pursuit of studies and careers in STEM (Wang and Degol, 2017). The second explanation has it that the gender gap originates from sociocultural factors, such as inequalities in the social and educational systems, and gender role stereotypes that determine explicit and implicit biases of boys and girls in how they perceive and evaluate their and others' performance and abilities in STEM (Charlesworth and Banaji, 2019; Girelli, 2022). For instance, exposure to role models, prior experiences with STEM and the expectations of others (e.g., parents) can contribute to the emergence of biased explicit and implicit (i.e., less conscious) beliefs on boys and girls abilities in STEM, which can influence individuals' behaviors, performance and learning experience (Miller and Halpern, 2014; Flore and Wicherts, 2015; Master et al., 2017; Charlesworth and Banaji, 2019), with possible long-lasting effects on girls' motivation to pursue studies or careers in the STEM domain (Charlesworth and Banaji, 2019). Master et al. (2021), for example, show how the stereotype that girls have lower interest in CS and Engineering than boys can cause gender disparities in motivation for CS education and in engaging in novel activities in this field. This link between stereotypes and interest in CS persists throughout high school, an age at which students typically make choices about their higher education. Thus, early elementary school can be a critical period to introduce children to counter-stereotypical examples, before stereotypes are firmly endorsed.

Recently, neuroscientific research has suggested that gender-related ability differences are actually the product of biopsychosocial interactions between biological predispositions and sociocultural experience (Miller and Halpern, 2014; Wierenga et al., 2019). In that interpretation, sex-differences in brain maturation can interact with sociocultural factors such as children's experiences, determining differences in cognitive performance in specific domains, like in language or spatial tasks (Miller and Halpern, 2014; Wierenga et al., 2019). These relative cognitive strengths or weaknesses may in turn affect students' perception to be able to perform STEM tasks and mediate the relationship between gender and task anxiety (Maloney et al., 2012), with possible consequences on students' motivation toward STEM.

Research shows that gender differences in basic cognitive skills underpinning STEM achievement can be observed from early childhood (Wang and Degol, 2017). However, as STEM gender-related stereotypes develop from children's experience of STEM activities and role models, they may emerge at a different age in different domains, depending on children's opportunity to be exposed to those activities and models. For instance, gender stereotypes on science and scientists do not seem to emerge before late primary school, because formal science instruction is sporadic in early grades of it (Miller et al., 2018). Likewise, gender-interest stereotypes about Engineering being more suited for boys are evident from grade 1, whereas in CS children seem to endorse gender-interest stereotypes only later, from grade 3 (Master et al., 2021).

All along child development, various sociocultural effects, among which the influence of stereotypes, may thus cause gender differences in STEM to appear that are not simply intrinsically sex-related, or associated to original predispositions. Master (2021) observe that gender stereotypes channeled via membership in social groups influence children's interest and motivation toward CS, their ability beliefs and their sense of belonging, which are prodromic to task avoidance and failure in STEM disciplines. As such effects may cause considerable distortion in the child's learning experience, it is important to examine the emergence of gender differences at early ages, when gender-ability stereotypes, i.e., the belief that boys are better at performing certain tasks than girls, are not yet strongly endorsed by children.

### Gender Differences in STEM

Studies exploring gender differences in STEM achievements show that they are most frequently observed in older students. Investigating the acquisition of mathematical abilities, Kersey et al. (2018) reports that boys and girls from 6 months to 8 years do not differ in early mathematical abilities. Stoet and Geary (2013) find them instead in favor of boys, at older age ranges, among higher-performing 15-year-old students. The latter finding seems to correlate with the observation that a lowering of self-efficacy beliefs in mathematics by girls and a parallel increase in boys occur between the fourth and the ninth grades (Reilly et al., 2019; Mejía-Rodríguez et al., 2020).

As noted, only a few studies address the issue of gender differences in CS as yet, in spite of the fact that women are extremely underrepresented in it, for education and career (Schmidt, 2011; Beyer, 2014; Denner et al., 2014; UNESCO, 2017). A widespread belief has it that male students have greater natural inclination to and ability with Information and Communication Technology, ICT (Jackson et al., 2008). This conjecture aligns with a meta-analysis in Cai et al. (2017), which shows boys in the age range between secondary school and college to have higher self-efficacy in ICT and more positive attitudes toward it than peer girls. These findings, however, measure bias-susceptible self-perception instead of actual skills: they may be predictive of attitude, but not of actual performance (Honicke and Broadbent, 2016). Moreover, those studies address a population of higher-education students likely exposed to well-structured and robust gender stereotypes. In younger children, these strong self-beliefs and gender-ability stereotypes may be not yet fully formed (Master et al., 2021). Although young children (from kindergarten to second grade education) already begin to form opinions about which technologies and tools would be better suited for boys and girls, gender attitudes toward technologies are still mild at this age (Sullivan and Bers, 2016). For instance, Master et al. (2017) report that children as young as 6 years already hold emergent gender stereotypes regarding computing, believing that boys should be more interested and better at coding and robotics than girls. The latter (gender-ability) beliefs however are less strong than the former (gender-interest) stereotypes (Master et al., 2021), and their effects on children's performance likely depend on children's prior experiences with digital technologies and coding (Gerson et al., 2022). Other findings seem to support the hypothesis of lesser influence of gender stereotypes in ICT activities at an early age. Aesaert and van Braak (2015) report primary school girls to have better technical ICT skills and higher-order ICT competences than boys. A subsequent meta-analysis corroborates that view by reporting girl-favoring gender differences in ICT literacy, with effect sizes larger in primary than secondary schools (Siddiq and Scherer, 2019).

### Gender Differences in Computational Thinking

Few studies zoom from broad ICT into the specifics of CS. When they do, they look for gender differences in coding as part of Computational Thinking (CT) activities. CT is a set of thinking skills, precursor of CS education, generally understood to comprise four constituents: (1) problem analysis via abstraction and decomposition, to distill core patterns from the original problem, to break it into smaller parts and systematically tackle each of them; (2) algorithmic thinking, to enable the development of predefined re-usable executable procedural tools for solving the given problem and classes of them; (3) evaluation of the outcomes of the solution plan, correcting it where it fails (also known as debugging), feeding all of that into (4) generalization, to lift problem-solving methods and solutions to application to similar problems (Wing, 2006; Resnick et al., 2009; Roman-Gonzalez et al., 2017; Shute et al., 2017; Yasar, 2017; Nardelli, 2019). Coding is a concrete way of practicing CT skills that consists in generating instructions (program's code) in a way that yields executable plans, whose effect to the problem can be empirically ascertained.

The studies that explored gender-related differences in CT or coding have produced contrasting findings. A study by Kožuh et al. (2018) reports finding no gender differences in problem solving skills involved in programming for fourth to sixth graders. Similarly, Papavlasopoulou et al. (2020), who used eye-tracking measures to assess the performance during coding workshops of 8- to 17-year-old students new to coding, a larger age range than Kožuh et al. (2018)'s study, report finding no statistically significant difference in gaze behaviors or learning gains between boys and girls. However, some qualitative gender-related differences emerged between girls and boys in the strategies used and in the perceptions of the coding activities. Price and Price-Mohr (2021), who explored the performance of 32 children between 10- and 11-year-old in an exercise aimed at animating stories with text-based coding, do not find gender differences in the process of coding or in the quality of the produced animations. Jiang and Wong (2021), find gender differences to be insignificant across fourth to sixth graders in the approach to conditionals, logical operators, pattern recognition, and generalization.

Other studies, involving older, fifth to tenth grade, students (Roman-Gonzalez et al., 2017; Statter and Armoni, 2017) have found significant gender differences in coding, although their findings are inconsistent regarding the direction of the gender effect. Statter and Armoni (2017) report results on seventh-grade students showing some advantage for girls in the learning of CS abstraction, and a greater effect of a learning intervention on girls, causing girls to regard CS as more than just programming, which boys did not. Conversely, Roman-Gonzalez et al. (2017) found a significant difference in CT tasks in favor of boys, with statistically significant differences emerging from grade 7, and a further increase of the gender gap between girls and boys in older, ninth to tenth grade, students. Also Yücel and Rizvanoglu (2019) found gender differences in coding among 11- to 14-year-old students, observing that they were associated with girls' lower self-confidence in performing the coding task and greater perception of task difficulty in comparison with boys. However, even in older, 14 to 19-year-old, high school students, gender differences in performance on coding tasks do not always emerge (Lau and Yuen, 2009).

As exposure to digital technologies and coding may significantly affect gender differences in perception, beliefs and motivations toward it (Master et al., 2017; Gerson et al., 2022), assessing children's coding skills as early as their first experience with coding becomes especially important. A recent systematic review (Bati, 2022) of experimental evidence on programming (i.e., coding applied to the creation of true computer programs), and CT in early childhood education, found that girls and boys from 3 to 5 years perform similarly in them.

To the best of our knowledge, however, only two studies have explored gender differences in CT and coding among children (4–7 years) exposed to it for the first time (Sullivan and Bers, 2013, 2016). Both studies report finding significant gender effects in favor of boys. The former study (Sullivan and Bers, 2013) found kindergarten boys to be better than girls in CT activities involving building with robotic materials. The latter (Sullivan and Bers, 2016), which focused on children aged 4 to 7 years, found similar performance across boys and girls in CT tasks involving basic coding skills, but a significantly better performance of boys in the use of more advanced coding constructs, such as repeat loops dependent on sensor readings.

A possible interpretation of these findings is that explicit or implicit gender stereotypes may affect actual performance even at a young age (Steele, 1997; Spencer et al., 1999). An alternative hypothesis, tested in this study, is that gender differences at this young age are the result of other influences, such as differences in the cognitive skills that underpin coding and perhaps CT in general (Halpern and LaMay, 2000; Miller and Halpern, 2014; Grissom and Reyes, 2019). As noted earlier, in young children approaching coding for the first time, gender stereotypes are not yet fully structured or endorsed and, if present, they have most likely mild effects (Martin et al., 1990; Sullivan and Bers, 2016). Thus, if gender differences in CT are observed at this early age, other (e.g., cognitive) factors could account for such differences. Our study tests this particular hypothesis, which was not explored in the cited works by Sullivan and Bers (2016). Where Sullivan and Bers (2016) use robotics on the grounds of it giving a playful and engaging touch to the learning ground, in the study presented in this paper we used the Code.org platform under similar premises. Much like Sullivan and Bers, we looked at whether the coding abilities of young (first-grade) children, all novice to coding, are affected by gender differences. The additional angle we brought into this study is to determine whether any such emerging gender differences associate with gender differences in children's executive functioning (EF), in particular planning and response inhibition, cognitive abilities closely related to problem-solving and CT (Arfé et al., 2019, 2020).

### Gender Differences in Executive Functioning

To date, the hypothesis that gender differences in coding, where they occur, can be mediated by gender differences in EF has not been tested yet. Executive functioning involves cognitive abilities used by individuals to focus on task, override automatic or impulsive responses and organize their behavior toward a goal. EF include response inhibition skills, working memory, switching, or the ability to flexibly adapt to different tasks, and more complex abilities like planning, which are involved in goal-directed behaviors and problem-solving (Miyake et al., 2000; Zelazo et al., 2003; Diamond, 2013; Viterbori et al., 2017). As noted earlier, coding tasks involve problem-solving processes that make significant demands on several levels of EF, including response inhibition, working memory (Shute et al., 2017; Di Lieto et al., 2020), and planning (Arfé et al., 2019, 2020).

When CT skills are practiced, the cited EF processes are also set in motion. Besides showing a strong association between coding abilities and first graders' planning skills (Arfé et al., 2019, 2020), between 5- and 6-year-old children's coding abilities and response inhibition (Arfé et al., 2019, 2020; Di Lieto et al., 2020), and between 5- and 6-year-old children's coding abilities and working memory (Di Lieto et al., 2020), prior research has also shown the existence of gender differences in the maturation of EF (Unterrainer et al., 2013; Grissom and Reyes, 2019; Wierenga et al., 2019). Although gender differences in executive functioning are not overwhelming (Grissom and Reyes, 2019), they are indeed observed in some domains, such as response inhibition and control over impulsive responses. For instance, males are found to be more impulsive and have more reduced reaction times than female (Grissom and Reyes, 2019). Inhibition and impulse control seem to mature earlier in girls than in boys. Indeed, between the age of 3 and 5, girls are reported to have better inhibition skills. Boys seem to catch up with girls only later, around the age of 6 (Klenberg et al., 2001). There also is empirical evidence that girls show better planning skills than boys during preschool years (Unterrainer et al., 2013) as well as that this advantage is maintained also during school years (Warrick and Naglieri, 1993; Naglieri and Rojahn, 2001). In a large-scale study involving 2,200 participants aged 5–7 to 11–17 years, Naglieri and Rojahn (2001) have shown a consistent advantage in planning skills for girls over boys across those age groups. The seemingly faster maturation of inhibition and planning in girls is of particular interest, as the ability to inhibit impulsive responses is an important prerequisite to an analytic approach to problem solving, and thus, by extension, to coding tasks. Likewise, planning is a core component skill of algorithmic thinking (the ability to define a sequence of steps to get to an objective) (Arfé et al., 2019, 2020). The existence of gender differences favoring girls in response inhibition and planning from as early as 5–6 years of age would cause expecting advantage for girls over boys to emerge also in the coding tasks that involve algorithmic thinking. Notably, this expectation goes in an opposite direction to what found by Sullivan and Bers (2016).

### The Study

The study presented in this paper explored the manifestation of gender differences in coding in young (5–7 year-old) children exposed to coding for the first time (goal 1), assessing whether any such observed differences were mediated by gender differences in planning or response inhibition, measured by standardized planning and inhibition tests (goal 2). Based on prior studies that demonstrate an association between coding skills and children's inhibition and planning abilities, we regarded inhibition and planning abilities as a potential mediator of the effects of gender on coding skills.

## Method

### Participants

One-hundred and nine first-graders aged from 5 to 7 years from schools located in northern Italy were enrolled in the study (45 girls, 41%, and 64 boys, 59%). Children had no prior experience with coding. They all took part in one-month introductory course to coding at the beginning of the school year, as part of a larger research project (Arfé et al., 2020).

The mean age of participants in this study was 6.00 for girls and 6.05 for boys. None of the participants had certified developmental disabilities or attentional problems. Demographic data are reported separately for girls and boys in Table 1. As children's prior exposure to digital technologies and parents' socioeconomic status can be associated to the development of coding abilities in children (Chiazzese et al., 2017; Gerson et al., 2022), these two factors were considered in this study. To assess such factors we used a short socio-demographic questionnaire that children's parents returned with written informed consent to participation in the study.

**Table 1:** Gender differences in age, SES (means, standard deviations, and *t*-test), and use of digital devices (Chi-square test).

	**Girls**		**Boys**			
	**(*****n*** **=** **45)**		**(*****n*** **=** **64)**			
**Variable**	***M***	***SD***	***M***	***SD***	***t***	**χ^2^**	***p***
Age	6	0.48	6.05	0.41	−0.55		0.59
SES	5.87	1.57	5.58	1.41	1.00		0.32
Computer						0.64	0.42
Tablet						0.05	0.81
Smartphone						0.73	0.39

*Socioeconomic status (SES)*: Children's socioeconomic status was estimated based on the level of education of the child's parents (both mother and father), on a scale from 0 (less than primary school) to 4 (college or above), and on the level of parents' occupation, from 1 (unemployed) to 4 (professional roles). A composite score was calculated as the non-weighed sum of the highest education and occupation score obtained by either parent (mother or father), with maximum score 8.

*Familiarity with technology*: This indicator was gauged by asking parents about children's daily use of personal computer, smartphone, or tablet devices in their home environment. The number of girls and boys that were reported to make daily of any such device was computed and compared by chi-square analyses.

The study was approved by the Ethical Committee of the Department of Developmental Psychology at the authors' institution.

### Study Design

Following Sullivan and Bers (2016), in this paper we examined children's ability to code at their first approach with coding, i.e., after an introductory course to coding at the beginning of grade one. Children's planning and inhibition skills were also assessed, and their mediation role in explaining gender differences in coding was tested. Coding, planning and response inhibition abilities were examined both *before* (time 1, T1) and *after* (time 2, T2) the coding course. This allowed to ascertain whether gender differences in coding or EF were present at any of those moments.

### Procedure and Materials

#### Coding Introductory Course

**Figure 1:**
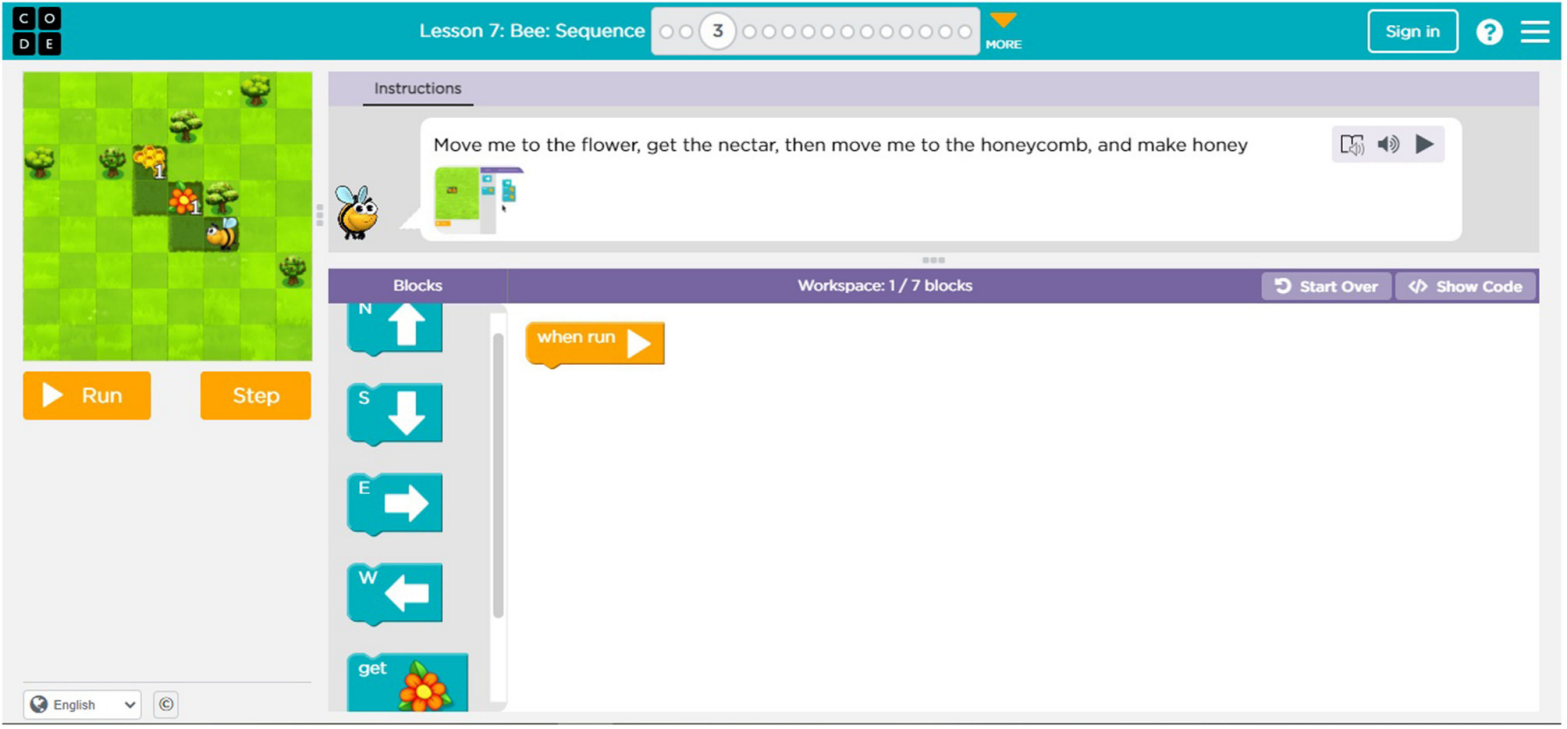
Lesson 7, course 1 (https://studio.code.org/s/course1/lessons/7/levels/3?lang=en-US) .

At the start of the school year all participants received a 1-month introductory course to coding through a selected choice of coding games from Code.org (https://code.org/). The coding environment and tasks proposed by the Code.org platform propose visual block-based programming tasks for beginners. Individual children write their code on that platform by moving code blocks from a toolbox panel into a programming panel, to generate code sequences (programs) whose execution should achieve predefined results. All coding games in Code.org involve the use of coding blocks to instruct a sprite (angry bird, bee, zombie) so that they can reach a target or perform expected actions. Visual and textual informative feedback is provided at every execution in order that children can easily monitor their progress on the screen. Likewise, programming errors are immediately visible to the child. The target not being reached manifests by the sprite crashing against a wall or failing to find a route to the target (see Figure 1). Children were progressively introduced to coding blocks of increasing logical difficulty, for example, from simple sequences to repetitions (loops).

Examining children's needs is crucial to the design of effective instructional coding activities (Ronsivalle et al., 2019). Since our participants were unfamiliar to coding as well they were beginning readers, the training was structured on course 1 of the Code.org platform “Programma il Futuro”^1^, the most basic and initial one. The children took part in coding lessons in the school's laboratory and always used a computer to carry out the assigned exercises. Although peer-based collaborative environments are thought to aid in the development of coding and CT skills (Flórez et al., 2017), the results of a pilot study suggested that the children were easily distracted by peers when working in pairs, and that the workload was not evenly shared. Classroom activities were thus designed to allow children to work individually at an assigned computer post in the school's laboratory. As children of this age are typically more familiar to touch-screen devices than to the use of mouse devices (Papadakis, 2021), a familiarization lesson took place before the course began, to accustom children to the use of the mouse. During the course, children were exposed to four main components of CT involved in coding (analysis of the problem space; decomposing problems; algorithmic thinking; evaluating and revising plans) and were allowed to practice with games that involved all such components. A group of post-graduate students (all female), trained to teach coding to children conducted each training session in collaboration with the first author of the study. During the lessons, the class teachers were present but did not intervene, except if children explicitly requested it. Postgraduate students, supported by the study's first author, were the same throughout the whole course. In turn, one post-graduate student led the lesson by explaining at the beginning of each new exercise the objective and functions to solve the problem. Other students supported the class and stood ready to answer questions from the children. One student was assigned the role of observer to point out any deflection from the planned protocol in each lesson. Such observers checked for example whether the support strategies were always the same and were balanced for each child, what difficulties did children encounter in carrying out the exercises, what function (such as repetition loop) was the hardest to learn. Children performed all games individually in the classroom and were requested to signal when they completed each task, to confront and discuss their solution. Group-wise corrections ended each exercise. The support students used scaffolding strategies to support children during both individual task performance and group correction. For example, they used questions-and-hints to stimulate children's approach to the solution of the problem at hand. All children received 60 min bi-weekly coding lessons for 4 weeks (eight lessons in total). Girls and boys received the same coding lessons and practiced coding by playing the same sequence of coding games. They took part in all coding lessons together. Participation in the coding course was not mandatory for children, who were free to withdraw at any time. Table 2 reports the full lesson plan. Children's actual liking of coding activities was not assessed through self-reports or systematic observations. However, all children appeared to be actively engaged and to enjoy the proposed activities thoroughly. No child asked to withdraw.

**Table 2:** Lessons plan. Selected coding games from programma il futuro, Course 1 (https://programmailfuturo.it/come/primaria/vecchie-lezioni-tecnologiche/corso-1).

**Coding sessions**	**Course 1**	**Trial number**	**Content**
Session 1	Lesson 3	1, 6	Jigsaw: Drag and drop
	Lesson 4	2, 5, 6, 7	Maze: Sequence
Session 2	Lesson 4	8, 10	Maze: Sequence
	Lesson 5	3, 4, 5, 6, 7	Maze: Debugging
Session 3	Lesson 8	4, 5, 6, 7, 8	Artist: Sequence
	Lesson 5	8, 9,10	Maze: Debugging
Session 4	Lesson 8	9, 10, 11	Artist: Sequence
	Lesson 10	4, 5, 6, 7, 8	Artist: Shapes
Session 5	Lesson 13	1, 2, 3, 4	Maze: Loops
	Lesson 13	5, 6, 7	Maze: Loops
Session 6	Lesson 13	8, 9, 10, 11, 12	Maze: Loops
Session 7	Lesson 14	3, 5, 6, 7, 8, 9	Bee: Loops
Session 8	Lesson 18	2, 4, 5, 6, 7	Artist: Loops
Closing session	Classroom discussion	What have we learned?	Metacognitive reflection on the goals of computational thinking and the meaning of programming

The data presented in this paper were collected as part of a larger study aimed to evaluate the effects of coding on children's EF. Thus, neither the instructors nor the children involved in the data collection were informed of the goal of the present study, that is to say, they did not know that gender differences in coding would also be assessed.

#### Assessment of Coding, Planning and Response Inhibition Skills

Children's coding, planning and response inhibition skills were assessed both before (T1) and after (T2) the coding lessons. Coding skills were assessed thorough children's ability to solve four coding games on Code.org. Two standardized neurocognitive tests, the Tower of London (Fancello et al., 2013), and a numerical Stroop test (Marzocchi et al., 2010) were used to assess their general planning and inhibition skills.

##### Coding Skills

Before starting the assessment, children were invited to practice with two coding games from Code.org, under guidance by the experimenter. The assessment started after the practice phase, in which the child familiarized with the Code.org platform and the mouse-based drag-and-drop mechanics necessary to perform the coding tasks. All children were asked to solve four coding problems individually and autonomously. To solve each coding trial, a maximum of three attempts were allowed, after which the trial was counted as failed. Specifically, the assessment involved solving trials 9 (lesson 4), 2 (lesson 5), 3 (lesson 8), 4 (lesson 14) from Code.org (Course 1, Italian platform, https://programmailfuturo.it/come/primaria/vecchie-lezioni-tecnologiche/corso-1). Trial 9 required guiding the Angry bird sprite to proceed in successive steps to reach a given target. Trial 2 involved debugging. Trial 3 required placing blocks in a sequence apt to instruct an artist sprite to draw a target geometric shape. Trial 4 consisted of using repetition loops. All of these exercises were of the same type as those in the coding introductory course.

For each trial, two scores were recorded as measures of children's coding skills:

(1) Accuracy: a score of 2 was given if the child successfully solved the problem at the first attempt, 1 when solving it at the second attempt, 0 otherwise;
(2) Time spent planning: the seconds elapsed from the moment the child was presented the trial to the moment s/he moved the first code block (which corresponded to starting to write the program). Time spent planning reflects the children's ability to plan their responses in advance and inhibit less mature trial-and-error strategies that are typical of younger children's approach to problem solving (Harter, 1930). Planning time was calculated on all trials, whether solved successfully or not, as in standardized planning measures (i.e., Tower of London, below). Planning time may reflect children's exploration of the coding platform, planning a sequence of steps for solving the coding problem, as well as pauses and hesitations (as, for example, holding the mouse) during the task.

##### Planning Skills

Planning ability was assessed by the Tower of London test (ToL; Luciana et al., 2009). In this study we used a version standardized for a population aged 4–13 years (Fancello et al., 2013).

The test requires the child to reproduce a configuration of three colored (blue, red, and green) small balls on three vertical sticks of different heights, according to a precise set of rules (e.g., moving one ball at a time; not holding the ball or placing it on the table, after picking it up). The entire test consists of 12 trials of increasing difficulty. All 12 trials were presented with no interruption criteria. As for the coding games, children's performance was scored for:

(1) Accuracy: each attempt was scored 1 if the child performed the trial correctly within 1 min, without breaking any rule; 0 otherwise.
(2) Planning time: counting from when the trial is shown to the child until when s/he makes the first move.

Reliability indices for this test are 0.57 for accuracy scores and 0.71 for planning times (Fancello et al., 2013).

##### Response Inhibition Skills

The Numerical Stroop test of the Batteria Italiana ADHD (BIA, Marzocchi et al., 2010) was used to assess children's ability to inhibit automatic responses. The Numerical Stroop test of the BIA, standardized for children aged 6–11, assesses response inhibition. The child is presented with a table that displays in each cell, from left to right, a digit from 1 to 5 (e.g., the digit 5), repeated n times (e.g., 3 times). The child is instructed to say as quickly and accurately as possible how many times the given digit (in the example, “5”) is shown in the cell (in the example, “three” times). To succeed in the task, the child must suppress automatic digit recognition (i.e., inhibiting the automatic response “5”). Performance is scored for:

(1) Accuracy: number of errors and self-corrections.
(2) Inhibition time: the seconds required to complete the task.

Test-retest reliability and validity are not provided by the manual. Arfé et al. (2020) report moderate test-retest reliability of this test for accuracy, *r* = 0.34, and adequate reliability for inhibition time, *r* = 0.62. Concurrent validity, computed by correlating the performance on the numerical Stroop and the NEPSY-II verbal response inhibition subtest, is *r* = 0.44 for accuracy and *r* = 0.48 for inhibition time.

Differences between girls and boys in age and SES were examined by independent-samples *t* tests. The different distribution between girls' and boys' use of digital devices (daily use of computer, smartphone, and tablet) was explored by chi-square tests.

The data analysis was performed in three steps.

1. Independent-samples *t* tests were used to test gender differences in coding, planning and response inhibition abilities before and after the coding lessons. Statistical significance was set as *p*-value < 0.05.
2. Pearson's correlations were run to explore the association between children's coding, planning (i.e., performance on the ToL), and response inhibition skills (performance on the Stroop task) before and after the introductory course to coding. Significant correlations between these abilities is indeed a condition necessary for assuming a mediation effect of children's planning and response inhibition skills on coding (Kraemer et al., 2008).
3. Mediation analyses were run to assess direct and indirect gender effects on children's coding abilities after the course (at T2). Mediation analyses allow exploring both direct and indirect effects of one variable (A) on another variable (B), where indirect effects refer to the underlying mechanism by which variable A influences variable B through a third (C) mediator variable (MacKinnon, 2008). In the present study, the use of mediation models allowed testing both the hypothesis of a direct influence of gender on children's coding ability (direct effect model) and the hypothesis that gender effects on coding are mediated by children's planning and inhibition skills (indirect effect models).

Mediation analyses were conducted using IBM SPSS Statistics 23.0 and Hayes' process model 4, with gender (dummy variable: girls or boys) as predictor, planning time or accuracy in coding after the course (T2) as criterion variables, and T2 planning time or accuracy on the Tower of London test (ToL), and T2 response inhibition time or errors on the Stroop test as mediators. SES and children's performance on coding, planning (ToL) and inhibition (Stroop) tasks before the course (at T1) were covariates. Confidence interval for each indirect effect was estimated considering both mediators and criterion variables at time 1 and at time 2 (95% confidence, 5000 bootstrap samples).

**Figure 2:**
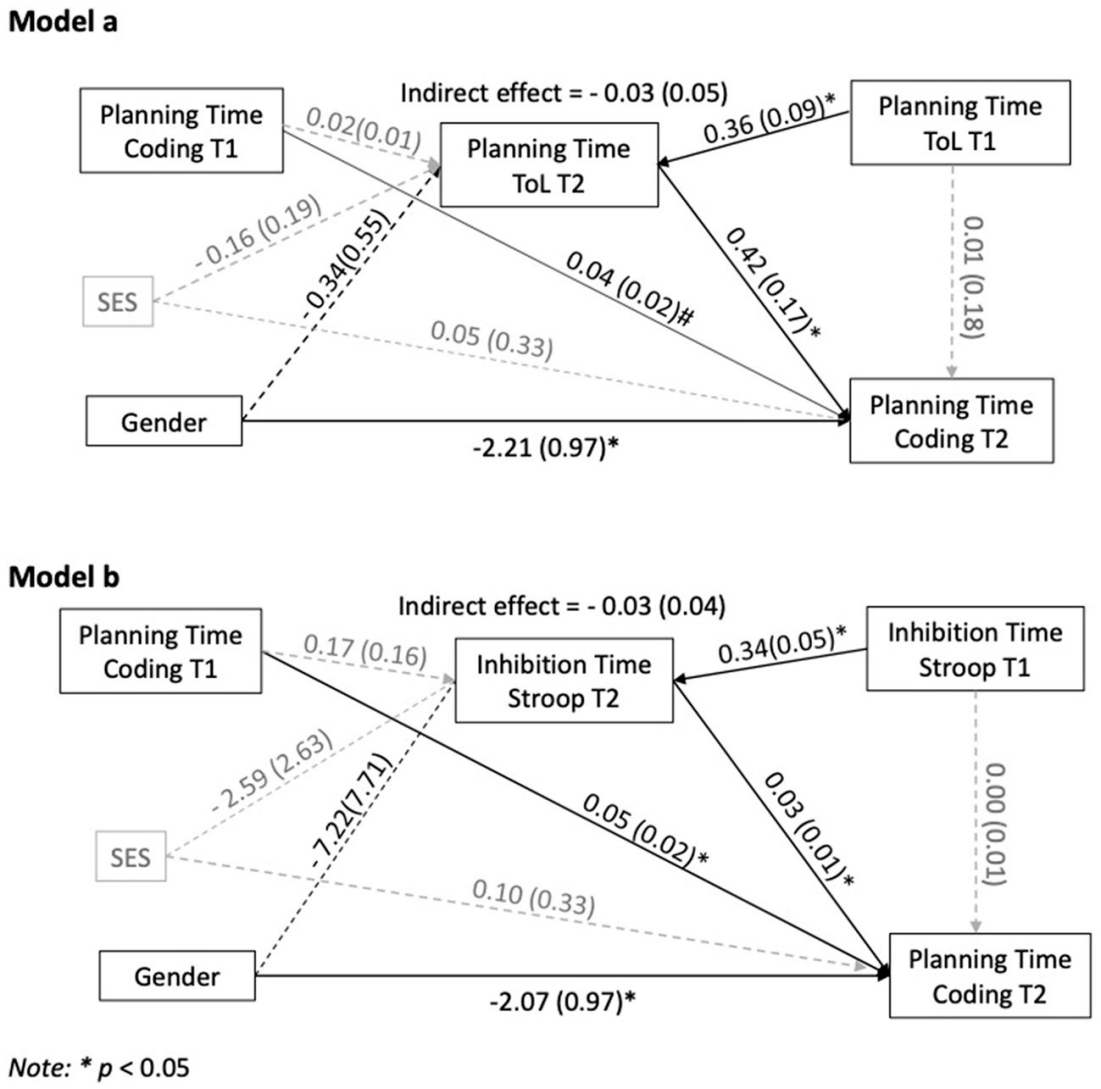
Mediation models. Gender effects on coding planning time. *p < 0.05.

**Figure 3:**
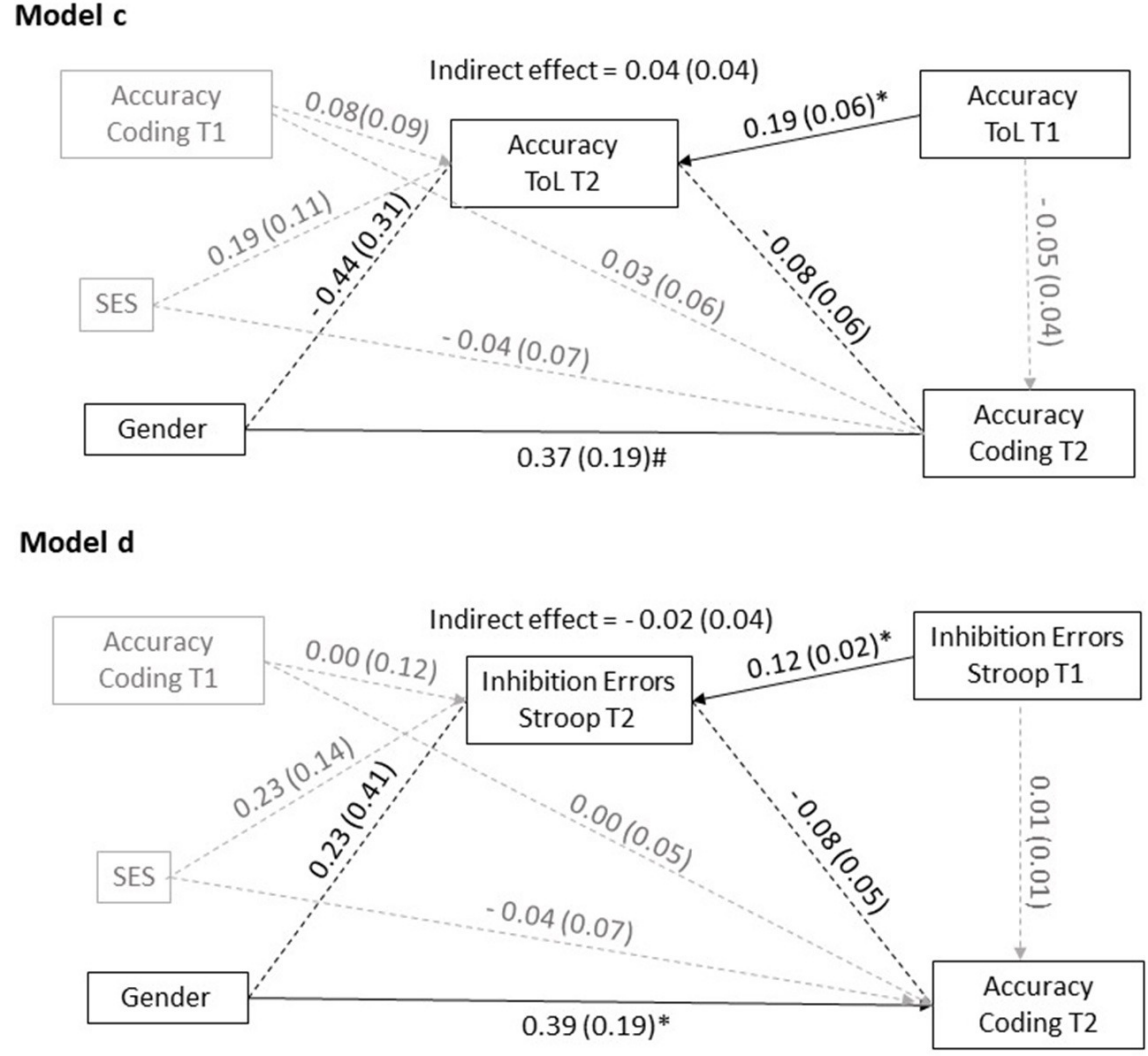
Mediation models. Gender effects on coding accuracy. #*p* = 0.05; *p < 0.05.

To examine mediation effects, four mediation models were tested: *Models a* and *b*, represented in Figure 2, assessed the direct and indirect effects of gender, the predictor variable, on children's planning time at coding games at T2, the outcome variable. In *Model a*, indirect effects were assessed considering the mediation of planning time on the ToL. Thus, T2 planning time on the ToL was the mediating variable (M). In *Model b*, the indirect effects were assessed by considering the mediating role of response inhibition time. Thus, T2 inhibition time on the Stroop test was the mediating variable. Similarly, *Models c* and *d*, in Figure 3, assessed the direct and indirect effects of gender (predictor) on children's accuracy in coding (outcome variable). In *Model c*, T2 accuracy on the ToL was the mediating variable. In *Model d*, errors in the Stroop test was the mediating variable. The covariates for all models were SES and the children's performance on coding, and planning (ToL) or inhibition (Stroop) tasks at T1. This arrangement allowed testing for the different impact of the learning experience for boys and girls, and to control for SES-related differences.

## Results

Between-group differences in age, SES, and familiarity with technology are reported in Table 1. The *t*-tests and chi-square analyses showed that the two groups were equivalent for age, *t*_(107)_ = −0.55, *p* = 0.59, SES, *t*_(107)_ = 1.00, *p* = 0.32, and familiarity with digital devices: use of computer, χ^2^ = 0.64, *p* = 0.42, tablet, χ^2^ = 0.05, *p* = 0.81, and smartphone, χ^2^ = 0.73, *p* = 0.39.

### Between-Group Differences in Coding, Planning and Response Inhibition

Table 3 reports the results of the between-group (girls, boys) comparisons in coding, planning and response inhibition abilities. After the introductory course to coding, significant differences in coding were observed. Girls spent significantly more time planning, *t*_(107)_ = 2.60, *p* = 0.01. However, their results were significantly less accurate, *t*_(107)_ = −2.12, *p* = 0.04, than those of boys. The effect sizes, reported in Table 3, are moderate. The performance of the two groups in coding *before* the coding course was equivalent. No significant differences emerged between girls and boys in planning or response inhibition, neither before nor after the coding course.

**Table 3:** Differences between girls and boys in coding, planning, and inhibition skills at time 1 (T1, Before) and Time 2 (T2, After) the coding course.

	**Girls**		**Boys**			
	**(*****n*** **=** **45)**		**(*****n*** **=** **64)**			
**Variable**	***M***	***SD***	***M***	***SD***	***t***	***p***	**Cohen's *d***
**Before the coding course (T1)**
Planning time coding	34.02	25.15	28.49	23.18	1.18	0.24	0.23
Accuracy coding	3.38	1.89	3.38	1.85	0.01	0.99	0.00
Planning time ToL	6.20	2.68	6.27	3.23	−0.13	0.90	−0.02
Accuracy ToL	5.38	2.74	5.77	2.75	−0.73	0.47	−0.14
Inhibition time Stroop	215.39	52.04	210.58	83.91	0.34	0.10	0.07
Inhibition errors Stroop	7.51	7.66	9.42	11.65	−0.96	0.25	−0.19
**After the coding course (T2)**
Planning time coding	11.44	5.02	8.82	5.28	2.60	0.01*	0.50
Accuracy coding	5.87	0.87	6.27	1.03	−2.12	0.04*	−0.41
Planning time ToL	6.68	2.55	6.30	3.40	0.64	0.52	−0.12
Accuracy ToL	9.36	1.65	8.94	1.79	1.24	0.22	0.24
Inhibition time Stroop	180.26	40.65	171.20	49.97	1.00	0.70	0.19
Inhibition errors Stroop	2.00	2.34	2.39	2.44	−0.84	0.93	−0.16

*p < 0.05; Planning time coding, Seconds spent planning on the coding games; Accuracy coding, Accuracy on the coding games; Planning time ToL, Seconds spent planning on the Tower of London test; Accuracy ToL, Accuracy on the Tower of London test; Inhibition time Stroop, Seconds required to complete the task on the Numerical Stroop test; Inhibition errors Stroop, Number of errors and self-corrections on the Numerical Stroop test.

### Associations Between Coding, Planning and Inhibition Skills

The correlational analyses, reported in Table 4, revealed a significant, although moderate, association between children's planning time in coding and planning time on the ToL both *before* (T1) the coding course, *r* = 0.30, *p* = 0.001, and *after* it (T2), *r* = 0.31, *p* = 0.001. Planning time in coding correlated significantly with response inhibition times *after* the coding course, *r* = 0.29, *p* < 0.005, but did not show significant associations with children's performance in response inhibition *before* the course.

**Table 4:** Means, standard deviations and intercorrelations between measures before and after the coding course.

**Variable**	**Before the coding course (T1)**						**After the coding course (T2)**					
	**1**	**2**	**3**	**4**	**5**	**6**	**1**	**2**	**3**	**4**	**5**	**6**
1 Planning time coding	1						1					
2 Accuracy coding	0.042	1					−0.346**	1				
3 Planning time ToL	0.302**	0.219	1				0.308**	−0.347**	1			
4 Accuracy ToL	0.308**	0.361**	0.448**	1			0.126	−0.20*	0.341**	1		
5 Inhibition time	0.072	−0.152	0.174	−0.046	1		0.287**	−0.264**	0.146	0.017	1	
6 Inhibition errors	−0.093	−0.249**	−0.172	−0.318**	0.235	1	0.273**	−0.116	−0.051	−0.172	0.329**	1
***M***	30.78	3.38	6.24	5.61	212.57	8.63	9.90	6.10	6.46	9.11	174.94	2.23
***SD***	24.06	1.85	3.00	2.74	72.22	10.2	5.31	0.98	3.07	1.74	46.37	2.40

*p < 0.05; **p < 0.01; Planning time coding, Seconds spent planning on the coding games; Accuracy coding, Accuracy on the coding games; Planning time ToL, Seconds spent planning on the Tower of London test; Accuracy ToL, Accuracy on the Tower of London test; Inhibition time, Seconds required to complete the task on the Numerical Stroop test; Inhibition errors, Number of errors and self-corrections on the Numerical Stroop test.

Accuracy scores on the coding games and the ToL were significantly associated both *before* the coding course (T1), *r* = 0.36, *p* < 0.001, and *after* the course (T2), *r* = −0.20, *p* = 0.04. The negative association between planning accuracy (ToL) and accuracy in coding at T2 indicates that after the coding course the children with a better performance on the ToL performed worse in coding. We shall return to this finding in the discussion. Accuracy on the coding games correlated significantly with inhibition errors *before* the course, at T1, *r* = **–**0.25, *p* < 0.01, and with response inhibition times *after* the course (T2), *r* = **–**0.26, *p* < 0.01.

### Mediation Analyses: Direct and Indirect Gender Effects on Coding Skills

Although no gender-related differences in planning and response inhibition emerged from the *t*-test analyses, it was still possible that children's planning and inhibition skills mediated the effects of gender on coding. The hypothesized mediation relationship was examined by testing the significance of the indirect effects of gender on coding planning time (*Models a* and *b*) and on coding accuracy (*Models c* and *d*) with ToL planning time or Stroop inhibition time (*Models a* and *b*) and accuracy on the ToL or on the Stroop test (*Models c* and *d*) as potential mediators.

The effect, whether direct or indirect, is considered significant if the interval between the upper and lower confidence bounds does not include zero. The four mediation models are reported in Figures 2, 3. Table 5 reports the direct, indirect and total model effects.

**Table 5:** Direct, indirect, and total effects of the mediation models.

**Mediator**	**Effect**	**Path**	**Point estimate**	**95% CI**	
				**Lower**	**Upper**
Planning time ToL	Direct effect	Gender → Planning time coding	−2.21*	−4.13	−0.29
		Gender → Planning time ToL	−0.34	−1.44	0.75
		Planning time ToL → Planning time coding	0.42*	0.08	0.76
	Indirect effect	Gender → Planning time ToL → Planning time coding	−0.15	−0.69	0.32
	Total effect	Gender → Planning time coding	−2.35*	−4.32	−0.39
Accuracy ToL	Direct effect	Gender → Accuracy coding	0.37	−0.01	0.75
		Gender → Accuracy ToL	−0.44	−1.06	0.18
		Accuracy ToL → Accuracy coding	−0.08	−0.20	0.04
	Indirect effect	Gender → Accuracy ToL → Accuracy coding	0.03	−0.05	0.13
	total effect	Gender → Accuracy coding	0.40*	0.03	0.78
Inhibition time	Direct effect	Gender → Planning time coding	−2.07*	−3.99	−0.15
		Gender → Inhibition time	−7.22	−22.50	8.07
		Inhibition time → Planning time coding	0.03*	0.00	0.05
	Indirect effect	Gender → Inhibition time → Planning time coding	−0.18	−0.60	0.31
	Total effect	Gender → Planning time coding	−2.25*	−4.20	−0.31
Inhibition errors	Direct effect	Gender → Accuracy coding	0.39*	0.02	0.77
		Gender → Inhibition errors	0.23	−0.58	1.05
		Inhibition errors → Accuracy coding	−0.08	−0.17	0.01
	Indirect effect	Gender → Inhibition errors → Accuracy coding	−0.02	−0.11	0.05
	Total effect	Gender → Accuracy coding	0.38*	0.00	0.76

*p < 0.05: Confidence interval for indirect effect was estimated with bootstrap method (95% confidence, 5,000 bootstrap samples). Planning time coding, Seconds spent planning on the coding games at T2 (after the course); Accuracy coding, Accuracy on the coding games at T2; Planning time ToL, Seconds spent planning on the Tower of London test at T2; Accuracy ToL, Accuracy on the Tower of London test at T2; Inhibition time, Seconds required to complete the task on the Numerical Stroop test at T2; Inhibition errors, Number of errors and self-corrections on the Numerical Stroop test atT2; CI, confidence interval.

All parameters are estimated controlling for SES, coding, planning and response inhibition (time and accuracy measures) at T1, before the coding course.

#### Gender Effects on Coding Planning Time

An inspection of Table 5 and of *Model a*, reported in Figure 2, shows that gender has direct effects on the time spent planning in the coding games at T2 (B = −2.21, *p* = 0.02). Planning time in coding tasks *before* the coding course (T1) has direct effects on children's planning time in coding *after* the course (T2, B = 0.04, *p* = 0.06). Planning time on the ToL at T2 is predicted by planning time on the ToL at T1 and has significant effects on planning time in coding at T2 (B = 0.42, *p* = 0.02). However, the indirect effect of gender through this mediator is not significant.

*Model b* (Figure 2), shows that gender and coding planning time *before* the coding course (T1) have direct effects on children's planning time in coding *after* the course (T2), respectively B = −2.07, *p* = 0.04 and B = 0.05, *p* = 0.02. Inhibition time *after* the course (T2) is predicted by inhibition time at T1, *before* the course, and has effects on planning time in coding at T2 (B = 0.03, *p* = 0.04). However, the indirect effect of gender through this mediator is insignificant (see also Table 5).

#### Gender Effects on Coding Accuracy

In *Model c*, reported in Figure 3, the direct effect of gender on coding accuracy at T2 approaches statistical significance (B = 0.37, *p* = 0.05). Planning accuracy on the ToL at T1 predicts accuracy on the ToL at T2 (B = 0.19, *p* < 0.01), which does not have significant effects on coding accuracy at T2. The indirect effect of gender through planning accuracy (ToL) is insignificant.

*Model d* shows a significant effect of inhibition errors at T1, *before* the course, on inhibition errors *after* the course (T2) (B = 0.12, *p* < 0.01), and a direct effect of gender on coding accuracy after the course (T2) (B = 0.39, *p* = 0.04). Again, the indirect effect of gender, through the mediator (inhibition errors at T2) is insignificant.

As shown in Figures 2 and 3, the covariates, T1 accuracy in coding and SES, do not account for significant variance in the mediator or in the outcome variable.

Overall, gender effects on children's coding emerged immediately after their first experience with coding; yet, although children's planning and response inhibition skills and coding skills were significantly related, gender effects on coding abilities were not mediated by children's planning or response inhibition skills.

## Discussion

The study presented in this paper was inspired by prior research (Sullivan and Bers, 2013, 2016) that reported gender differences in children's coding to emerge since very early experience with it. In this study we further tested the hypothesis that gender differences may already exist among early-age (5–7 year-old) children at their first experience with coding. Moreover, we investigated whether any such gender differences were mediated by gender differences in cognitive abilities underpinning CT and coding.

There is evidence that girls and boys differ in the maturation of some cognitive functions that are known to be involved in coding: in particular, impulsive responses inhibition and planning (Warrick and Naglieri, 1993; Klenberg et al., 2001; Naglieri and Rojahn, 2001; Unterrainer et al., 2013; Grissom and Reyes, 2019). Yet, no studies have directly tested the hypothesis that the gender differences observed between girls and boys in coding could be related to differences in these underlying cognitive abilities. The original contribution of this study was to address this research question.

Prior research conducted with young children has shown that girls develop response inhibition skills and planning skills earlier than boys (Klenberg et al., 2001; Naglieri and Rojahn, 2001; Unterrainer et al., 2013). However, these gender-related developmental differences in executive functioning did not appear in our study. In contrast to our expectations and to the extant literature (Naglieri and Rojahn, 2001; Unterrainer et al., 2013), we did not find differences in planning between girls and boys on the ToL test. Indeed, boys and girls performed equally well on the ToL (planning), and on the Stroop task (response inhibition). Participants in our study ranged in age between 5 and 7 years, with a mean age of 6.03. It may be that at this age, and with school entrance, gender differences in response inhibition and planning have been leveled already (Klenberg et al., 2001; Unterrainer et al., 2013). In contrast with that, however, we did find significant gender differences in the coding tasks *after* the coding course, both in accuracy and planning time. Girls spent significantly more time planning on the coding tasks, without however achieving better performance. Conversely, they were significantly less accurate than boys in them. These gender differences emerged only *after* children experienced coding activities in class: No significant differences were indeed observed between boys and girls *before* the coding course.

The results of the mediation analyses clarified that the gender differences observed in coding *after* the course were not mediated by children's planning abilities (i.e., performance on the ToL) or response inhibition skills (i.e., performance on the Stroop task). Remarkably, the mediational analyses accounted for the effects of covariates like SES, and performance on the coding and cognitive tasks (planning and response inhibition) before the course. Thus, the observed differences cannot be attributed to these factors either.

Some studies have suggested that gender differences in coding may be due to the different strategies or approach to coding problems characteristic of girls and boys (e.g., Sullivan and Bers, 2016), which could be also related to a different use of children's own cognitive abilities. While these findings confirm boys' advantage observed in coding by Sullivan and Bers (Sullivan and Bers, 2013, 2016), they do not support the hypothesis that these differences are accounted for by cognitive predispositions to coding. We can hypothesize that sociocultural factors, such as gender-ability stereotypes, may have affected children's performance. Master et al. (2017) showed that first graders may already have embraced stereotypes that boys are better at programming than girls. If present in our participants, these stereotypes did not affect their performance at their pretest, but did so after the instructional experience with coding. That is, they were likely induced by this early experience and instructional activity. This observation contrasts with Master et al. (2017) finding that introducing girls to programming at this early age may induce counter-stereotypical beliefs and higher self-efficacy for programming. A difference between our study and Master et al.'s study is however that in Master et al.'s study children performed the programming activities in solo sessions, where boys and girls interacted individually with the experimenter. In this study, instead, coding activities were performed in the classroom, thus exposing children to making implicit or explicit comparisons among their performance, with differential effects on boys' and girls' self-confidence. We return on this hypothesis in the conclusion.

It is particularly worrying that the boys' advantage in performance on coding tasks emerged after the children were exposed to counter-stereotypical role models by being shepherded by (young) female trainers, observers, assistants, and experimenters. Other studies have shown that STEM gender stereotypes from early childhood to adolescence are not always influenced by the opportunity to interact with counter-stereotypical educators (McGuire et al., 2020). The mechanisms through which counter-stereotypical models may affect children's self-perception and performance in coding can be thus more complex. They could for instance depend on the nature of the interaction between children and the counter-stereotypical models: e.g., how much children are engaged with them, which activities are performed by these counter-stereotypical models, which is their attitude toward girls and boys. These factors could be the focus of future investigations.

Some more words should be spent on the negative association we found between children's accuracy in coding and the ToL after the coding course. This finding was unexpected, particularly considering that the same two measures were significantly and positively associated *before* the coding course. Although the statistical tests did not reveal significant differences between boys and girls in the performance on the planning task (ToL), girls scored slightly better than boys after the course and spent slightly more time planning on the ToL. However, their performance on the coding tasks was lower than for boys. This may suggest that they made a worse use of their cognitive resources than boys, in what would amount to a gender-related effect. Indeed, by-group correlations reveal that the association between coding and planning skills, albeit non-significant, is negative only for girls.

Planning time and accuracy in coding at T2 were also negatively correlated. Girls spent significantly more time planning in the coding tasks, and yet – as noted above – their accuracy was lower than that of boys. The longer time spent by girls in planning aligns with the findings of other studies, confirming a tendency of girls to be less impulsive and to control more their responses than boys (Grissom and Reyes, 2019). In this study however, while boys and girls showed equivalent response inhibition skills, girls showed a lower coding performance than boys at T2. This observation suggests an alternative explanation: the longer time spent planning in the coding tasks by girls could reflect hesitations in planning more than greater control over their act. Such interpretation would align with findings showing that girls have lower self-confidence in performing coding tasks and greater perception of task difficulty in comparison with boys (Yücel and Rizvanoglu, 2019), which is associated to gender biases. Although the participants in our study were much younger than the participants in Yücel and Rizvanoglu's study, early emerging gender-ability beliefs could have influenced their perception of the coding tasks and, consequently, their performance (Master et al., 2021).

## Conclusions

The results replicated those of prior studies (Sullivan and Bers, 2016), showing that boy-favoring gender differences in coding can emerge at early age (5–7 years). Remarkably, gender differences in coding do not seem to be mediated by differences in cognitive abilities (e.g., planning or response inhibition) and emerged only after children had experienced coding in their classroom. This finding is particularly worrying on account of the young age of our study participants and of the fact that the two groups had equal experience of technological devices and similar cognitive abilities (i.e., similar planning and inhibition skills). Moreover, all of the personnel that conducted this study and interacted with the children in the training and the experiments were female, providing role models that should have been especially motivating (or at least reassuring) for girls. A possible interpretation of the gender effects observed in this study is that boys and girls matured different self-confidence in coding or different beliefs about it in consequence of their coding experience *in the classroom*. Although performing all games individually, children were requested to signal when they completed each task, to confront and discuss their solution. This individual working format coupled with classroom discussion of the individual outcomes may have stimulated a competitive approach to the coding task, which is often present in classrooms when learning tasks are performed individually and results are compared class-wide. Sullivan and Bers (2016) suggest that competitive learning environments could favor boys. In summary, boys could have been more motivated by interpreting the coding tasks as a competition, whereas girls could have been negatively influenced by this social environment. Different results could have emerged if we requested boys and girls to work in teams and collaboratively (see Sullivan and Bers, 2016).

### Limitations of The Study

The data presented in this study were collected as part of a larger project focused on the cognitive effects of coding. In the project we did *not* consider relevant sociocultural factors, such as children's implicit or explicit gender-related beliefs, which may have had significant influence on their performance in coding. In hindsight we have learned that future studies in this direction should explore the influence of motivational and sociocognitive variables (e.g., self-efficacy beliefs) and their interaction with children's cognitive abilities. We are taking this lesson learned home in the design of future interventions directed to digging further into this important gender-difference problem space.

A second limitation of this study is the lack of a qualitative analysis of children's strategies in solving the coding games. As demonstrated by other studies, gender differences could emerge in the way girls and boys approach the coding task (Sullivan and Bers, 2016). Exploring such differences and how they relate to children's cognitive abilities and the type of instruction children have received is important to inform the design of a coding curriculum. Children can be introduced to coding through individual or group activities, playing with robots and tangible environments or via virtual environments (as in our study). The way coding activities are structured, the instrument used, the social partners involved (peers or experts, within gender or across genders) could influence the way children approach coding, the coding skills they develop and the ideas they form about their coding skills (i.e., their self-efficacy beliefs). A more qualitative and finer-grained analysis of children's performance would be possible with the use of additional methodological tools, such as interviews, self-reports, or even behavioral observations and learning analytics. We are working in this very direction at the time of this writing.

A third and final limitation of the study concerns the lack of a fine-grained analysis of children's engagement in the coding course and of their relationship with digital technology. Engagement is known to be an important component of learning, which may be affected by self-perception over and above ability. Measuring engagement before, during, after the intervention should shed more light on the reasons for the emergence of the surprising results from this research. By the token, measuring familiarity with technologies calls for a finer-grained spectrum than merely considering how often children use digital devices, or which digital devices they use, examining also *how* they use them, for what goal, learning or leisure, in which way, alone and unassisted or with friends or seniors. Acquiring and analyzing such additional information would help gain a better understanding of children's prior experience with digital technologies in the way to anticipating engagement. The dimensions of engagement entail a social element and a learning element. Both are very important aspects of a wider study into the nature, origin, and mitigations of gender differences in relation to coding and, prospectively to Computer Science.

## Data Availability Statement

The raw data supporting the conclusions of this article will be made available by the authors, without undue reservation.

## Ethics Statement

The studies involving human participants were reviewed and approved by Ethical Committee for the Psychological Research of the University of Padova. Written informed consent to participate in this study was provided by the participants' legal guardian/next of kin.

## Author Contributions

BA: conception and design of the project, project coordination, paper drafting and revising, and data interpretation. CM: data gathering, coordination of the data gathering, article drafting, and data analyses. LR: guiding the data analysis. TV: article drafting and revising and coordination of the study. All authors contributed to the article and approved the submitted version.

## Conflict of Interest

The authors declare that the research was conducted in the absence of any commercial or financial relationships that could be construed as a potential conflict of interest.

## Publisher's Note

All claims expressed in this article are solely those of the authors and do not necessarily represent those of their affiliated organizations, or those of the publisher, the editors and the reviewers. Any product that may be evaluated in this article, or claim that may be made by its manufacturer, is not guaranteed or endorsed by the publisher.

## Footnotes

^1^ https://programmailfuturo.it/come/primaria/vecchie-lezioni-tecnologiche/corso-1
“Programma il futuro” is an initiative of the Ministry of Education, University and Research (MIUR) in collaboration with the Consorzio Interuniversitario Nazionale per l'Informatica (CINI), aimed at giving schools a series of tools to provide students with the scientific and cultural bases of computer science.

## References

[B1] Aesaert K. van Braak J. (2015). Gender and socioeconomic related differences in performance based ICT competences. Comput. Educ. 84, 8–25. 10.1016/j.compedu.2014.12.017

[B2] Alonso M. T. Barba-Sánchez V. López Bonal M. T. Macià H. (2021). Two perspectives on the gender gap in computer engineering: from secondary school to higher education. Sustainability 13, 10445. 10.3390/su131810445

[B3] Anderson N. Lankshear C. Timms C. Courtney L. (2008). ‘Because it's boring, irrelevant and I don't like computers': Why high school girls avoid professionally-oriented ICT subjects. Comput. Educ. 50, 1304–1318. 10.1016/j.compedu.2006.12.003

[B4] Arfé B. Vardanega T. Montuori C. Lavanga M. (2019). Coding in primary grades boosts children's executive functions. Front Psychol. 10, 2713. 10.3389/fpsyg.2019.02713 31920786 PMC6917597

[B5] Arfé B. Vardanega T. Ronconi L. (2020). The effects of coding on children's planning and inhibition skills. Comput. Educ. 148, 103807. 10.1016/j.compedu.2020.10380731920786

[B6] Bati K. (2022). A systematic literature review regarding computational thinking and programming in early childhood education. Educ. Inf. Technol. 27, 2059–2082. 10.1007/s10639-021-10700-2

[B7] Beyer S. (2014). Why are women underrepresented in Computer Science? Gender differences in stereotypes, self-efficacy, values, and interests and predictors of future CS course-taking and grades. Comput. Sci. Educ. 24, 153–192. 10.1080/08993408.2014.963363

[B8] Cai Z. Fan X. Du J. (2017). Gender and attitudes toward technology use: A meta-analysis. Comput. Educ. 105, 1–13. 10.1016/j.compedu.2016.11.00335801233

[B9] Charles M. Harr B. Cech E. Hendley A. (2014). Who likes math where? Gender differences in eighth-graders' attitudes around the world. Int. Studies Sociol. Educ. 24, 85–112. 10.1080/09620214.2014.895140

[B10] Charlesworth T. E. S. Banaji M. R. (2019). Gender in science, technology, engineering, and mathematics: issues, causes, solutions. J. Neurosci. 39, 7228–7243. 10.1523/JNEUROSCI.0475-18.2019 31371423 PMC6759027

[B11] Chiazzese G. Fulantelli G. Pipitone V. Taibi D. (2017). “Promoting computational thinking and creativeness in primary school children,” in Proceedings of the 5th International Conference on Technological Ecosystems for Enhancing Multiculturality. p. 1–7. 10.1145/3144826.3145354

[B12] Cvencek D. Meltzoff A. N. Greenwald A. G. (2011). Math–gender stereotypes in elementary school children. Child Develop. 82, 766–779. 10.1111/j.1467-8624.2010.01529.x 21410915

[B13] Denner J. Werner L. O'Connor L. Glassman J. (2014). Community college men and women: a test of three widely held beliefs about who pursues computer science. Commun. College Rev. 42, 342–362. 10.1177/0091552114535624

[B14] Di Lieto M. C. Pecini C. Castro E. Inguaggiato E. Cecchi F. Dario P. Cioni G. Sgandurra G. (2020). Empowering executive functions in 5- and 6-year-old typically developing children through educational robotics: An RCT study. Front. Psychol. 10, 3084. 10.3389/fpsyg.2019.03084 32116879 PMC7012808

[B15] Diamond A. (2013). Executive functions. Ann. Rev. Psychol. 64, 135–168. 10.1146/annurev-psych-113011-143750 23020641 PMC4084861

[B16] Fancello G. S. Vio C. Cianchetti C. (2013). ToL, Torre di Londra. Test di Valutazione delle Funzioni Esecutive (Pianificazione e Problem Solving). Trento: Erickson.

[B17] Fisher A. Margolis J. (2003). “Unlocking the clubhouse: Women in computing,” in Proceedings of the 34th SIGCSE Technical Symposium on Computer Science Education. p. 23. 10.1145/611892.611896

[B18] Flore P. C. Wicherts J. M. (2015). Does stereotype threat influence performance of girls in stereotyped domains? A meta-analysis. J. School Psychol. 53, 25–44. 10.1016/j.jsp.2014.10.002 25636259

[B19] Flórez B. F. Casallas R. Hernández M. Reyes A. Restrepo S. Danies G. (2017). Changing a generation's way of thinking: teaching computational thinking through programming. Rev. Educ. Res. 87, 834–860. 10.3102/0034654317710096

[B20] Frieze C. (2005). Diversifying the images of computer science: Undergraduate women take on the challenge! ACM SIGCSE Bull. 37, 397–400. 10.1145/1047124.1047476

[B21] Gerson S. A. Morey R. D. van Schaik J. E. (2022). Coding in the cot? Factors influencing 0–17s' experiences with technology and coding in the United Kingdom. Comput. Educ. 178, 104400. 10.1016/j.compedu.2021.104400

[B22] Girelli L. (2022). What does gender has to do with math? Complex questions require complex answers. J. Neurosci. Res. 10.1002/jnr.25056 35443070

[B23] Gnambs T. (2021). The development of gender differences in information and communication technology (ICT) literacy in middle adolescence. Comput. Human Behav. 114, 106533. 10.1016/j.chb.2020.106533

[B24] Grissom N. M. Reyes T. M. (2019). Let's call the whole thing off: evaluating gender and sex differences in executive function. Neuropsychopharmacology 44, 86–96. 10.1038/s41386-018-0179-5 30143781 PMC6235899

[B25] Halpern D. F. LaMay M. L. (2000). The smarter sex: a critical review of sex differences in intelligence. Educ. Psychol. Rev. 12, 229–246. 10.1023/A:1009027516424

[B26] Harter G. L. (1930). Overt trial and error in the problem solving of preschool children. Pedagogical Semin. J. Genetic Psychol. 38, 361–372. 10.1080/08856559.1930.105322706235749

[B27] Honicke T. Broadbent J. (2016). The influence of academic self-efficacy on academic performance: A systematic review. Educ. Res. Rev. 17, 63–84. 10.1016/j.edurev.2015.11.00234837413

[B28] Jackson L. A. Zhao Y. Kolenic A. Fitzgerald H. E. Harold R. Von Eye A. (2008). Race, gender, and information technology use: the new digital divide. CyberPsychol. Behav. 11, 437–442. 10.1089/cpb.2007.0157 18721092

[B29] Jiang S. Wong G. K. W. (2021). Exploring age and gender differences of computational thinkers in primary school: A developmental perspective. J. Computer Assisted Learning. 38, 60–75. 10.1111/jcal.12591

[B30] Kersey A. J. Braham E. J. Csumitta K. D. Libertus M. E. Cantlon J. F. (2018). No intrinsic gender differences in children's earliest numerical abilities. Npj Sci. Learn. 3, 1–10. 10.1038/s41539-018-0028-7 30631473 PMC6220191

[B31] Klenberg L. Korkman M. Lahti-Nuuttila P. (2001). Differential development of attention and executive functions in 3- to 12-year-old finnish children. Develop. Neuropsychol. 20, 407–428. 10.1207/S15326942DN2001_6 11827096

[B32] Kožuh I. Krajnc R. Hadjileontiadis L. J. Debevc M. (2018). Assessment of problem solving ability in novice programmers. PLoS ONE 13, e0201919. 10.1371/journal.pone.0201919 30208039 PMC6135368

[B33] Kraemer H. C. Kiernan M. Essex M. Kupfer D. J. (2008). How and why criteria defining moderators and mediators differ between the baron and kenny and macarthur approaches. Health Psychol. 27, S101–S108. 10.1037/0278-6133.27.2(Suppl.).S101 18377151 PMC3376898

[B34] Lau W. W. F. Yuen A. H. K. (2009). Exploring the effects of gender and learning styles on computer programming performance: Implications for programming pedagogy. Br. J. Educ. Technol. 40, 696–712. 10.1111/j.1467-8535.2008.00847.x

[B35] Luciana M. Collins P. F. Olson E. A. Schissel A. M. (2009). Tower of London performance in healthy adolescents: the development of planning skills and associations with self-reported inattention and impulsivity. Develop. Neuropsychol. 34, 461–475. 10.1080/87565640902964540 20183711 PMC4203700

[B36] MacKinnon D. P. (2008). Introduction to Statistical Mediation Analysis. Routledge.

[B37] Maloney E. A. Waechter S. Risko E. F. Fugelsang J. A. (2012). Reducing the sex difference in math anxiety: The role of spatial processing ability. Learn. Individual Differ. 22, 380–384. 10.1016/j.lindif.2012.01.001

[B38] Martin C. L. Wood C. H. Little J. K. (1990). The development of gender stereotype components. Child Develop. 61, 1891–1904. 10.2307/1130845 2083503

[B39] Marzocchi G. M. Re A. M. Cornoldi C. (2010). *BIA*. Batteria Italiana per l'ADHD. Trento: Erickson.

[B40] Master A. (2021). Gender Stereotypes Influence Children's STEM Motivation. Child Develop. Perspect. 15, 203–210. 10.1111/cdep.1242430564179

[B41] Master A. Cheryan S. Moscatelli A. Meltzoff A. N. (2017). Programming experience promotes higher STEM motivation among first-grade girls. J. Exper. Child Psychol. 160, 92–106. 10.1016/j.jecp.2017.03.013 28433822

[B42] Master A. Meltzoff A. N. Cheryan S. (2021). Gender stereotypes about interests start early and cause gender disparities in computer science and engineering. Proc. Nat. Acad. Sci. 118, e2100030118. 10.1073/pnas.2100030118 34810255 PMC8640926

[B43] McGuire L. Mulvey K. L. Goff E. Irvin M. J. Winterbottom M. Fields G. E. Hartstone-Rose A. Rutland A. (2020). STEM gender stereotypes from early childhood through adolescence at informal science centers. J. Appl. Develop. Psychol. 67, 101109. 10.1016/j.appdev.2020.101109 32255884 PMC7104893

[B44] Mejía-Rodríguez A. M. Luyten H. Meelissen M. R. M. (2020). Gender differences in mathematics self-concept across the world: an exploration of student and parent data of TIMSS 2015. Int. J. Sci. Mathemat. Educ. 19, 1229–1250. 10.1007/s10763-020-10100-x

[B45] Miller D. I. Halpern D. F. (2014). The new science of cognitive sex differences. Trends Cogn. Sci. 18, 37–45. 10.1016/j.tics.2013.10.011 24246136

[B46] Miller D. I. Nolla K. M. Eagly A. H. Uttal D. H. (2018). The development of children's gender-science stereotypes: a meta-analysis of 5 decades of U.S. Draw-A-Scientist Studies. Child Develop. 89, 1943–1955. 10.1111/cdev.13039 29557555

[B47] Miyake A. Friedman N. P. Emerson M. J. Witzki A. H. Howerter A. Wager T. D. (2000). The unity and diversity of executive functions and their contributions to complex ‘Frontal Lobe' tasks: A latent variable analysis. Cogn. Psychol. 41, 49–100. 10.1006/cogp.1999.0734 10945922

[B48] Naglieri J. A. Rojahn J. (2001). Gender differences in planning, attention, simultaneous, and successive (PASS) cognitive processes and achievement. J. Educ. Psychol. 93, 430–437. 10.1037/0022-0663.93.2.430

[B49] Nardelli E. (2019). Do we really need computational thinking? Commun. ACM 62, 32–35. 10.1145/3231587

[B50] Papadakis S. (2021). The impact of coding apps to support young children in computational thinking and computational fluency. a literature review. Front. Educ. 6, 657895. 10.3389/feduc.2021.657895

[B51] Papavlasopoulou S. Sharma K. Giannakos M. N. (2020). Coding activities for children: Coupling eye-tracking with qualitative data to investigate gender differences. Comput. Human Behav. 105, 105939. 10.1016/j.chb.2019.03.003

[B52] Price C. B. Price-Mohr R. (2021). Exploring gender differences in primary school computer programming classes: A study in an English state-funded urban school. Education 3–13, 1–14. 10.1080/03004279.2021.1971274

[B53] Reilly D. Neumann D. L. Andrews G. (2019). Investigating Gender Differences in Mathematics and Science: Results from the 2011 Trends in Mathematics and Science Survey. Res. Sci. Educ. 49, 25–50. 10.1007/s11165-017-9630-6

[B54] Resnick M. Maloney J. Monroy-Hernandez A. Rusk N. Eastmond E. Brennan K.. (2009). Scratch: Programming for all. Commun. ACM 52, 60–67. 10.1145/1592761.1592779

[B55] Roman-Gonzalez M. Perez-Gonzalez J. C. Jimenez-Fernandez C. (2017). Which cognitive abilities underlie computational thinking? Criterion validity of the computational thinking test. Comput. Hum. Behav. 72, 678–691. 10.1016/j.chb.2016.08.047

[B56] Ronsivalle G. B. Boldi A. Gusella V. Inama C. Carta S. (2019). How to implement educational robotics' programs in italian schools: a brief guideline according to an instructional design point of view. Technol. Knowl. Learn. 24, 227–245. 10.1007/s10758-018-9389-5

[B57] Schmidt F. L. (2011). A theory of sex differences in technical aptitude and some supporting evidence. Perspect. Psychol. Sci. 6, 560–573. 10.1177/1745691611419670 26168377

[B58] Shute V. J. Sun C. Asbell-Clarke J. (2017). Demystifying computational thinking. Educ. Res. Rev. 22, 142–158. 10.1016/j.edurev.2017.09.00325824671

[B59] Siddiq F. Scherer R. (2019). Is there a gender gap? A meta-analysis of the gender differences in students' ICT literacy. Educ. Res. Rev. 27, 205–217. 10.1016/j.edurev.2019.03.007

[B60] Spearman J. Watt H. M. G. (2013). “Women's Aspirations Towards “STEM” Careers,” in W. Patton (Ed.), *Conceptualising Women's Working Lives: Moving the Boundaries of Discourse*. Rotterdam: SensePublishers. p. 175–191. 10.1007/978-94-6209-209-9_10

[B61] Spencer S. J. Steele C. M. Quinn D. M. (1999). Stereotype Threat and Women's Math Performance. J. Exper. Soc. Psychol. 35, 4–28. 10.1006/jesp.1998.1373

[B62] Statter D. Armoni M. (2017). “Learning abstraction in computer science: a gender perspective,” in Proceedings of the 12th Workshop on Primary and Secondary Computing Education. p. 5–14. 10.1145/3137065.3137081

[B63] Steele C. M. (1997). A threat in the air: How stereotypes shape intellectual identity and performance. Am. Psychol. 52, 613–629. 10.1037/0003-066X.52.6.613 9174398

[B64] Stoet G. Geary D. C. (2013). Sex differences in mathematics and reading achievement are inversely related: within- and across-nation assessment of 10 years of PISA data. PLOS ONE 8, e57988. 10.1371/journal.pone.0057988 23516422 PMC3596327

[B65] Sullivan A. Bers M. U. (2013). Gender differences in kindergarteners' robotics and programming achievement. Int. J. Technol. Design Educ. 23, 691–702. 10.1007/s10798-012-9210-z

[B66] Sullivan A. Bers M. U. (2016). Girls, Boys, and Bots: Gender Differences in Young Children's Performance on Robotics and Programming Tasks. J. Inf. Technol. Educ. 15, 145–165. 10.28945/3547

[B67] UNESCO (2017). Cracking the code: Girls' and women's education in science, technology, engineering and mathematics (STEM)—UNESCO Digital Library. Available online at: https://unesdoc.unesco.org/ark:/48223/pf0000253479 (accessed August 23, 2022).

[B68] Unterrainer J. M. Ruh N. Loosli S. V. Heinze K. Rahm B. Kaller C. P. (2013). Planning Steps Forward in Development: In Girls Earlier than in Boys. PLOS ONE 8, e80772. 10.1371/journal.pone.0080772 24312240 PMC3842368

[B69] Viterbori P. Traverso L. Usai M. C. (2017). The role of executive function in arithmetic problem-solving processes: A study of third graders. J. Cogn. Develop. 18, 595–616. 10.1080/15248372.2017.1392307

[B70] Wang M. T. Degol J. L. (2017). Gender gap in science, technology, engineering, and mathematics (STEM): current knowledge, implications for practice, policy, and future directions. Educ. Psychol. Rev. 29, 119–140. 10.1007/s10648-015-9355-x 28458499 PMC5404748

[B71] Warrick P. Naglieri J. (1993). Gender differences in planning, attention, simultaneous, and successive (PASS) cognitive processes. J. Educ. Psychol. 85, 693–701. 10.1037/0022-0663.85.4.693

[B72] Wierenga L. M. Bos M. G. N. van Rossenberg F. Crone E. A. (2019). Sex effects on development of brain structure and executive functions: greater variance than mean effects. J. Cogn. Neurosci. 31, 730–753. 10.1162/jocn_a_01375 30726177

[B73] Wing J. M. (2006). Computational thinking. Commun. ACM 49, 33–35. 10.1145/1118178.1118215

[B74] Yasar O. (2017). The essence of computational thinking. Comput. Sci. Eng. 19, 74–82. 10.1109/MCSE.2017.3151241

[B75] Yücel Y. Rizvanoglu K. (2019). Battling gender stereotypes: A user study of a code-learning game, “Code Combat,” with middle school children. Comput. Human Behav. 99, 352–365. 10.1016/j.chb.2019.05.029

[B76] Zelazo P. D. Müller U. Frye D. Marcovitch S. Argitis G. Boseovski J. Chiang J. K. Hongwanishkul D. Schuster B. V. Sutherland A. (2003). The development of executive function in early childhood. Monographs Soc. Res. Child Develop. 68, vii−137. 10.1111/j.0037-976X.2003.00261.x 14723273

[B77] Zweben S. Aspray W. (2004). Undergraduate enrollments drop; department growth expectations moderate. *Computing Research News*. Available online at: http://archive.cra.org/Activities/workshops/broadening.participation/cra/Taulbee/2002-2003.pdf (accessed August 23, 2022).

